# Widely Targeted Metabolomics Analysis of Soybean and Chickpea and Their Different Advantages and New Functional Compounds for Diabetes

**DOI:** 10.3390/molecules27165297

**Published:** 2022-08-19

**Authors:** Pengshou Li, Yumiao Bian, Mengdan Li, Lingmei Li, Baosheng Zhao, Qixiang Ma, Yingchun Song, Jiuyi Li, Gangsheng Chen

**Affiliations:** 1 School of Food and Drug, Luoyang Normal University, Luoyang 471934, China; 2 Beijing Academy of Traditional Chinese Medicine, Beijing University of Chinese Medicine, Beijing 100029, China; 3 Cancer Institute, Fudan University Cancer Hospital and Cancer Metabolism Laboratory, Institutes of Biomedical Sciences, Fudan University, Shanghai 200032, China

**Keywords:** chickpea, soybean, widely targeted metabolomics, differential metabolites, diabetes

## Abstract

Soybean is widely used as a kind of bean for daily consumption. Chickpea is increasingly utilised because of its good healthcare function. At present, using chickpeas could have better results than soybeans in some areas. Previous studies of the two legumes focused on certain components and failed to fully reveal the differences between the two legumes. Thus, understanding the comprehensive similarities and differences between the two legumes is necessary to apply and develop these legumes effectively. In this study, we performed a UPLC-ESI-MS/MS-based widely targeted metabolomics analysis on two legumes. A total of 776 metabolites (including primary metabolites and secondary metabolites) were detected, which were divided into more than a dozen broad categories. The differential analysis of these metabolites showed that there were 480 metabolites with significant differences in relative contents between the two legumes. Compared with soybean, the expression of 374 metabolites of chickpea was down-regulated and that of 106 metabolites was up-regulated. The metabolic pathway analysis showed significant differences in the flavonoids biosynthesis, phenylpropanoid biosynthesis, linoleic acid metabolism and alkaloid biosynthesis between the two legumes. The advantages and applicability of the two kinds of legumes were confirmed through the analysis of anti-diabetic components. Moreover, some novel compounds (with contents higher than that of soybean) with hypoglycaemic activity were found in chickpea. This study provides an important reference for the in-depth study and comparative application of soybean and chickpea.

## 1. Introduction

Chickpea (*Cicer arietinum* L.) is a member of the genus *Cicer* in the *Leguminosae* sp., which originated in western Asia and along the Mediterranean coast [1]. A study showed that the chickpea production of the world was 15.08 million tons, thereby becoming the world’s third largest bean [2]. Chickpea is widely distributed in the Xinjiang region of China because of its strong drought resistance and tolerance [3]. Xinjiang Uygur Autonomous Region in China usually eats high-fat, high-calorie foods, such as beef and mutton, dairy products and high-sugar melons and fruits, but not many people suffer from diabetes and cardiovascular and cerebrovascular diseases. The United Nations Health Organization found that chickpeas played a key role in a balanced diet in their staple food, that is, grasp rice mixed with chickpeas, thus preventing diabetes and cardiovascular and cerebrovascular diseases [4]. Chickpea is also rich in protein, vitamins, carbohydrates, minerals, dietary fibre and other nutrients [5]. In addition, chickpea contains the following physiological functions: anti-osteoporosis, blood sugar reduction, cholesterol regulation, anti-oxidation and anti-cancer [6,7,8,9,10,11]. Chickpea has high healthcare value and remarkable development potential. At present, more than 20 active compounds of flavonoids, saponin, polysaccharide and organic acids had been isolated from chickpea [12,13]. However, these studies did not reveal the full extent of the identification and quantification of most metabolites in chickpea. Thus, the application and development of chickpea were markedly restricted.

Soybean originated from China and was derived from USSURI soybean; it has been planted in China for 5000 years [14]. Soybean not only contains protein, fat, vitamins, minerals and other basic nutrients but also phospholipids, sterols, isoflavones, saponins, oligosaccharides, vitamin E and other bioactive components [15]. Isoflavone substances, soy peptides and other substances had anti-oxidation, anti-cancer, prevention of osteoporosis, hypoglycaemic, cardiovascular risk reduction, oestrogen effect and other functional activities [16,17,18,19,20,21,22].

Soybeans and chickpeas are produced in large quantities, thus increasing opportunities to eat and use them in daily life. Consequently, a growing number of researchers are focusing on the strengths and weaknesses of chickpeas and soybeans to make them healthier to use in everyday life [23,24,25,26,27]. In addition, both legumes have been shown to have beneficial effects on diabetes, but studies of their metabolites were inadequate, so studies of their active components tended to focus only on certain isoflavones and triterpenoid saponins, the study had strong limitations [12,13,28]. Therefore, a comprehensive study of metabolites from soybean and chickpea and the differences in metabolites are very necessary to facilitate the effective use of the two legumes.

Widely targeted metabolomics analysis is a rapid and reliable approach for the detection of a wide range of plant metabolites [29]. In this study, widely targeted metabolomics analysis based on ultrahigh performance liquid chromatography-electrospray ionization-tandem mass spectrometry (UPLC-ESI-MS/MS) was used to analyse the metabolites in soybean and chickpea and the differences in metabolites between the two legumes. The metabolic pathways, which led to the differences in metabolic products, were also studied and analysed. The treatment of diabetes-related metabolites analysis revealed their differences in diabetes prevention and treatment. The comprehensive analysis of metabolic products provided an important reference for further research on the effective components of soybean and chickpea and the mechanism of their activities. A comprehensive understanding of the soybean and chickpea was obtained through this study, which also laid a solid foundation for their further research and application.

## 2. Results

### 2.1. Sample Quality Control Analysis

**Figure 1:**
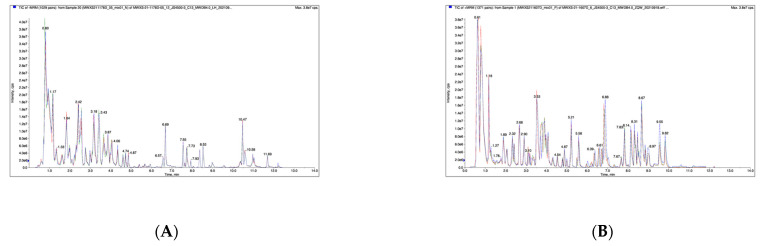
TIC overlapping figures of different QC samples (different colors represent different QC samples). (**A**) Negative ion mode. (**B**) Positive ion mode.

The overlapping display analysis of Total Ion Chromatography (TIC) of different QC samples (Figure 1A,B) confirmed the good repeatability of metabolites extraction and detection.

**Figure 2:**
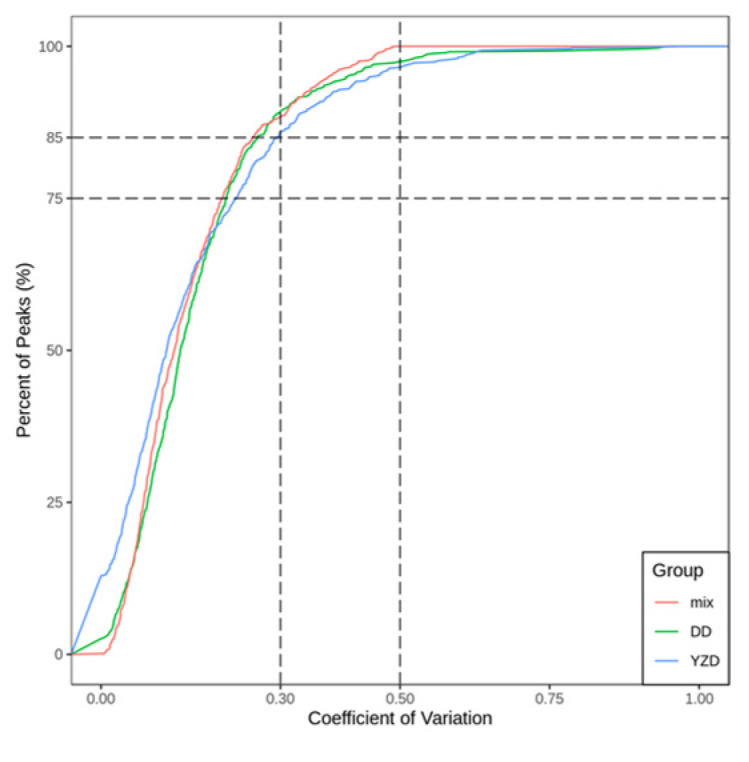
The CV distribution of each group.

A high proportion of the substance with a low CV value denotes the stability of experimental data. The proportion of substances with CV less than 0.5 was higher than 85%, which indicated that the experimental data was stable. The proportion of substances with CV less than 0.3 was higher than 75%, which indicated that the experimental data was remarkably stable. The CV distribution of each group (Figure 2) showed that the experimental data were remarkably stable.

### 2.2. Widely Targeted Metabolomics Analysis in Chickpea and Soybean

**Figure 3:**
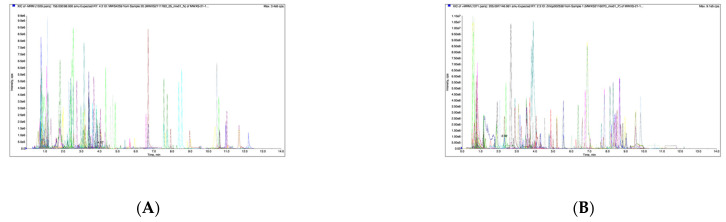
Multi-peak figure for MRM metabolite detection (each different colored mass spectrum peak represents one metabolite detected). (**A**) Negative ion mode. (**B**) Positive ion mode.

**Figure 4:**
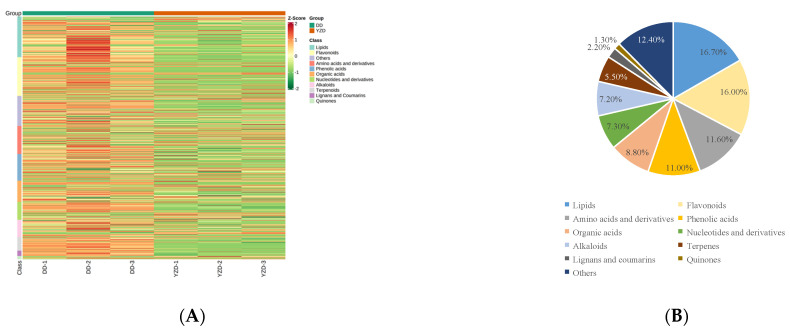
Metabolites in DD and YZD. (**A**) Heatmap of the metabolites in DD and YZD. The colour indicates the level of accumulation of each metabolite, from low (green) to high (red). The Z-score represents the deviation from the mean by standard deviation units. (**B**) Types and proportions of the identified metabolites from DD and YZD.

The primary and secondary metabolites of the two legumes were analysed by widely targeted LC-MS/MS to study the chemical constituents of chickpea and soybean (Figures S1 and S2). The relative contents of metabolites were analysed by the MRM mode using MultiQuant software (Figure 3A,B). A total of 776 metabolites, including more than 10 classes, were detected (Table S1 and Figure 4A,B). Some of these classes could be further divided into several secondary classifications (Table 1).

### 2.3. Multivariate Analysis of Identified Metabolites

**Figure 5:**
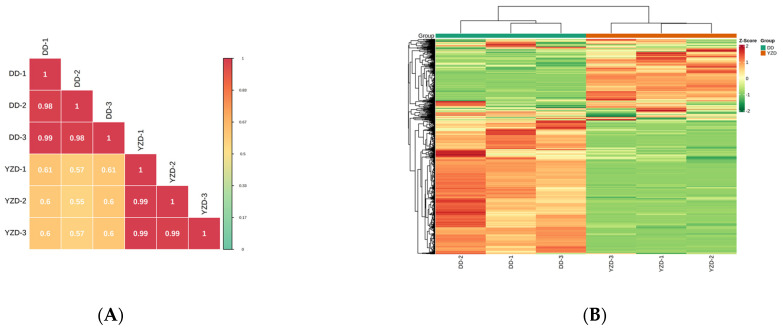

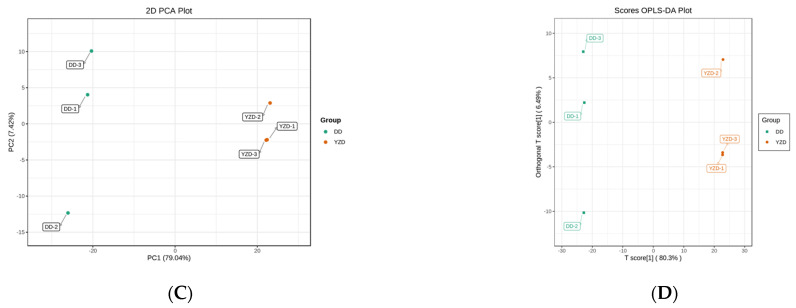
Multivariate analysis of identified metabolites. (**A**) Pearson’s correlation coefficients among the DD and YZD samples. (**B**) Hierarchical cluster analysis of the identified metabolites from the DD and YZD samples. (**C**) PCA of YZD and DD. (**D**) OPLS-DA of YZD and DD.

Multivariate analysis was used to further evaluate metabolites detected in YZD and DD. Correlation analysis showed that the correlation coefficients between the intra-group samples were close to 1 and the biological duplication was remarkably good (substantially higher than the inter-group samples correlation coefficients), which indicated that the obtained differential metabolites were reliable. The correlation coefficients between the YZD and DD groups also showed that YZD and DD had some biological duplications (Figure 5A). The data of metabolite content were normalized (Unit Variance Scaling, UV Scaling), and the accumulation pattern of metabolites in different samples was analysed by Hierarchical Cluster Analysis (HCA). According to the analysis results, the metabolites in YZD and DD samples could be clearly divided into two groups. The two groups of metabolites in YZD and DD samples were significantly different (Figure 5B).

The two groups of samples were analysed by Principal Component Analysis (PCA) to observe the variability in inter- and intra-group samples. The degree of variation in inter- and intra-group samples was studied (Figure 5C) by compressing the original data and taking PC1 (79.04% interpretation rate of data set) as the first principal component and PC2 (7.42% interpretation rate of data set) as the second principal component. Three biological duplications in DD group were concentrated on the left side of the PCA figure, and the distribution of the second principal component was scattered. By contrast, three biological duplications in YZD group were concentrated on the right side of the PCA figure, and the distribution of the second principal component was relatively concentrated. OPLS-DA evaluation of the variation degree in inter- and intra-group samples showed significant differences in metabolites between DD and YZD groups (Figure 5D and Figure S3).

### 2.4. Differential Metabolites between DD and YZD

**Figure 6:**
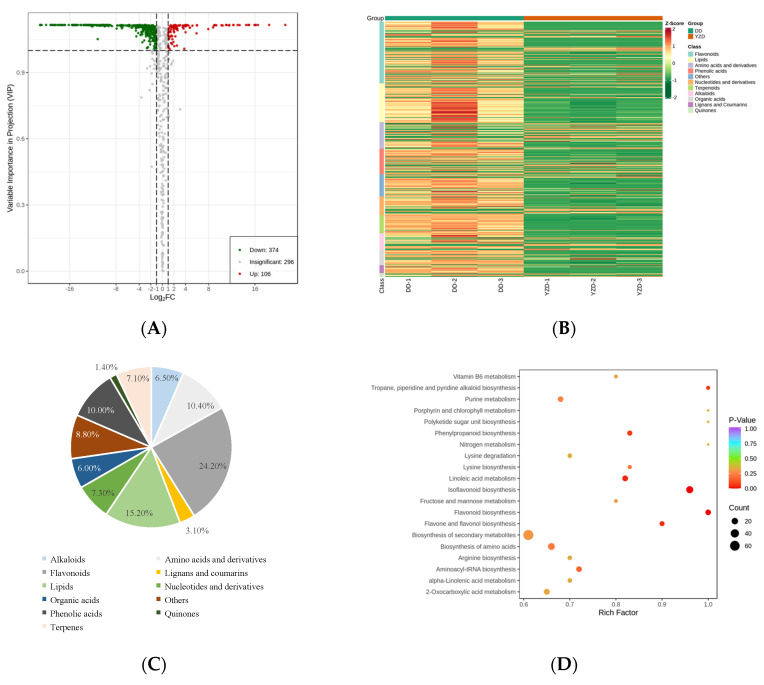
Analysis of DAMs between YZD and DD. (**A**) Volcano plot of the DAMs. (**B**) Heatmap of the DAMs. (**C**) Types and proportions of the DAMs. (**D**) Enrichment of KEGG pathways of DAMs.

The VIP values of OPLS-DA models and fold change were used to screen the differentially accumulated metabolites (DAMs). The screening conditions were as follows: fold change ≥ 2 or fold change ≤ 0.5, and VIP ≥ 1. A total of 480 DAMs were obtained by screening between DD and YZD (Table S2). Compared with DD, 374 putative metabolites were down-regulated and 106 putative metabolites were up-regulated in YZD (Figure 6A). These DAMs could be divided into more than 10 categories (Figure 6B). Most of these DAMs were concentrated in four types of compounds, including flavonoids (24.20%), lipids (15.20%), amino acids and derivatives (10.40%) and phenolic acids (10.00%, Figure 6C).

Through analysis, we got the differences of primary metabolites and the differences of secondary metabolites in YZD and DD (Table S3). For primary metabolites, 42 metabolites were up-regulated and 146 metabolites were down-regulated in YZD compared with DD. For secondary metabolites, 62 metabolites were up-regulated and 218 metabolites were down-regulated in YZD compared with DD. Compared with DD, YZD has 2 up-regulated metabolites and 10 down-regulated metabolites in other types of compounds. For primary and secondary metabolites, YZD had approximately 22% up-regulated metabolites and approximately 78% down-regulated metabolites compared with DD.

The KEGG database was used to map metabolites to metabolic pathways. A total of 298 metabolites were mapped in 80 metabolic pathways, including 174 DAMs (Table S4). The results of KEGG mapping of DAMs were classified in accordance with the type of pathway in KEGG, and most of the pathways focused on metabolism. By contrast, only a few focused on genetic and environmental information processing (Figure S4). According to these results, the KEGG pathway enrichment was analysed. The major enrichment of KEGG pathways included isoflavonoid biosynthesis, flavonoid biosynthesis, flavone and flavonol biosynthesis, phenylpropanoid biosynthesis, linoleic acid metabolism, and tropane, piperidine and pyridine alkaloid biosynthesis (Figure 6D and Table S4).

### 2.5. Analysis of Anti-Diabetic Components

Chickpeas and soybeans had anti-diabetic effects [30,31,32]. This study focused on lipids, saccharides, isoflavones, triterpene saponins and other metabolic products associated with diabetes. As shown in Figure 6B and Table 2, the contents of most lipids and saccharides from chickpeas were substantially lower than that of soybeans. The contents of most isoflavones and triterpenoid saponins in soybeans were higher than that in chickpeas.

Previous studies revealed that isoflavones, triterpenoid saponins and resistant starch were the main hypoglycaemic active components in chickpea [12,33,34]. This study also found many other types of compounds in chickpeas that had a hypoglycaemic effect. The relative contents of these compounds in chickpeas were higher than those in soybeans (Table S2). These compounds included amino acids and derivatives, such as L-glutamine [35,36], L-lysine [37], L-homoarginine [38], and phenolic acids, such as gallic acid [39,40], and so on. In addition, considering the hydrolysis reaction of glycosides in vivo, corresponding aglycones could be produced. We also analysed the glycosides which had good blood glucose lowering activity of their aglycones. These compounds included flavones such as acacetin, acacetin-7-O-galactoside, acacetin-7-O-glucoside [41,42,43], chrysoeriol-7-O-(6′′-malonyl) glucoside [44]; flavanones such as diosmetin-7-O-glucoside [45]; flavonols such as isorhamnetin-3-O-(6′′-malonylglucoside), isorhamnetin-3-O- (6′′-acetylglucoside), isorhamnetin-3-O-neohesperidoside [46,47], kaempferol-3-O- sambubioside, kaempferol-3-O-rutinoside, kaempferol-3-O-neohesperidoside, kaempferol-3-O-(2-O-Xylosyl-6-O-Rhamnosyl)Glucoside, kaempferol-3-O-(6′′′′-malonyl) sophorotrioside [48,49,50]; flavonoid carbonoside such as orientin-2′′-O-galactoside [51]. In addition to the considerable difference in the contents of biochanin A and its glycosides between chickpea and soybean, which had good hypoglycaemic activity, some compounds with hypoglycaemic effect in chickpeas that were far more abundant than in soybeans were also found (e.g., gallic acid, acacetin and its glycosides, isorhamnetin glycosides and chrysoeriol-7-O-(6′′-malonyl) glucoside). These compounds were far more abundant in chickpeas than in soybeans, and some were found only in chickpeas.

## 3. Discussion

Soybeans and chickpeas are common legumes in production and life. Chickpea, as a kind of plant with medicine and food homology, had good healthcare function [33]. An increasing number of studies had focused on chickpeas as a substitute for soybeans, such as for some functional foods for human beings and animal feed, to produce improved results [24,25,26,27,52,53,54]. Therefore, a comprehensive analysis of whether these types of legumes could be used as substitutes or their respective advantages and disadvantages is necessary for their further development and application. Thus, a comprehensive study of the metabolites from the two legumes and the differences in their metabolites is crucial. In this study, the primary and secondary metabolites of soybeans and chickpeas were comprehensively identified and relatively quantitative analysis was performed using widely targeted metabolomics analysis, involving more than a dozen kinds of metabolites. The results of this study provided important basic data for further research and development of soybeans and chickpeas.

A total of 776 metabolites were identified. In addition to lipids and amino acids and derivatives, we found that flavonoids, a major kind of secondary metabolites, were very abundant, even more abundant than amino acids and derivatives. Flavonoids is a kind of active compounds in nature, which would be beneficial to the development and application of two kinds of beans. Multivariate analysis of identified metabolites showed that there was less variability among samples in the chickpea group than in the soybean group. This indicated that the accumulation of metabolites was more stable during the growing process of chickpea. The 480 metabolites showed significant difference between soybean and chickpea by differential metabolites analysis. The identification and relatively quantitative analysis of these metabolites compensated for the lack of previous studies. The relative contents of 106 metabolites in chickpea were up-regulated compared with that in soybean. These metabolites of up-regulation may be the material bases for good healthcare function of chickpea. Like biochanin A, there is little in soybeans. The research and development of these material bases could be deepened. In addition, we were concerned that the two legumes because of the differences of growth habits and resistance to the natural environment, the corresponding metabolites were also different. For example, maackiain, a phytoalexin, was found in chickpeas but not soybeans. The relative contents of 374 metabolites in soybean were up-regulated compared with that in chickpea. Among them, primary metabolites provided rich nutrients, such as lipids and amino acids. The relative contents of most secondary metabolites were also up-regulated and this could make it a source of some pharmacodynamic material basis.

Chickpeas and soybeans could be used as good ingredients to prevent diabetes in the diet. The above study revealed numerous differences in their metabolites. This finding suggested that the application of the two kinds of beans as supplementary food for blood sugar reduction should be emphasised. The potential diabetes prevention and treatment of soybean and chickpea were also discussed on the basis of identified metabolites. Chickpea is more suitable for diabetes prevention and adjuvant treatment of the modern population due to its low lipids and saccharides contents. For the isoflavones and triterpenoid saponins, although higher content in soybean, was less effective in reducing blood sugar due to its higher lipids. The results of this study could also explain why people with chickpea as an auxiliary food could be a good control of diabetes and cardiovascular diseases in Xinjiang Uygur Autonomous Region in China and other places rich in beef and mutton (high fat) as well as a variety of fruits (high sugar). In addition, some isoflavones, such as biochanin A, trifolirhizin and prunetin-5-O-glucoside, which demonstrated high contents in chickpea, could be studied and developed for their hypoglycaemic activity. The compounds related to the hypoglycaemic activity of higher contents in chickpea than in soybean were also analysed, which provided a new reference for studying the hypoglycaemic mechanism of chickpea. Soybean may be able to play an effective role in blood sugar reduction via lipid removal.

The enrichment of DAMs by KEGG pathway revealed differences between the two legumes in the metabolic pathways of isoflavonoid biosynthesis, flavonoid biosynthesis, flavone and flavonol biosynthesis, phenylpropanoid biosynthesis, linoleic acid metabolism and tropane, piperidine and pyridine alkaloid biosynthesis. Among them, flavonoid, phenylpropanoids, and alkaloids demonstrated biological activities. In addition, some differences were observed in the metabolic pathways of purine metabolism, biosynthesis of amino acids, and aminoacyl-tRNA biosynthesis. These differences could also have an impact on health.

## 4. Materials and Methods

### 4.1. Plant Materials

**Figure 7:**
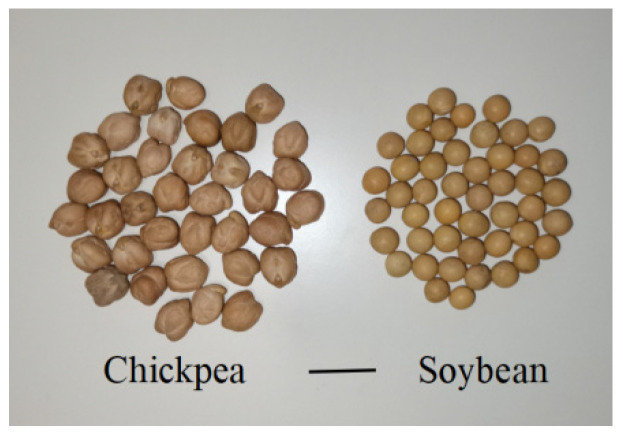
Chickpea and Soybean. Scale bar = 1 cm.

Chickpeas (Muying No.1) and soybeans (Heihe 43) were used in this study (Figure 7). These two varieties have the characteristics of wide planting range, good performance, high yield and the good representation. Chickpeas were collected from Mori Kazakh Autonomous County, Xinjiang, China (43.837396 N, 90.296538 E; representative planting area) on August 2021. Chickpea was sown with 45 cm row spacing and 13 to 15 cm plant spacing and approximately 75,000 plants per hectare. Soybeans were collected from Heihe Soybean Association, Heihe, Heilongjiang, China (50.241354 N, 127.536307 E; representative planting area) on September 2021. There were 4 rows of 100 cm ridge and about 12 cm plant spacing and approximately 400,000 plants per hectare.

### 4.2. Sample Preparation and Metabolite Extraction [55,56]

The chickpea group was named YZD and the soybean group was named DD. Each group was set up with three biological replicates, each of which was collected from five different plants. Therefore, the YZD-1, YZD-2 and YZD-3 in the chickpea group and DD-1, DD-2 and DD-3 in the soybean group were observed. Metabolite extraction process: (1) The samples were placed in a lyophilizer (Scientz-100F, Scientz, Ningbo, China) for vacuum freeze drying. (2) After freeze-drying, the samples were prepared into powder by a grinder (MM 400, Retsch, Shanghai, China) for 1.5 min at 30 Hz. (3) The 100 mg sample powder was dissolved in 1.2 mL 70% methanol. (4) The samples were swirled every 30 min for 30 s (total vortex six times) and then the samples were kept in a refrigerator at 4 °C overnight. (5) The supernatant was extracted and filtered by microporous membrane (SCAA-104, 0.22 μm pore size; ANPEL, Shanghai, China) after centrifugation (12,000 rpm, 10 min) and stored in the sample bottle for UPLC-MS/MS analysis.

### 4.3. Quality Control Measurements

The quality control (QC) samples comprised equal-weight chickpea samples mixed with soybean samples and used to analyse the repeatability of the mass spectrometric results under the same treatment. In the instrumental analysis process, one QC sample was inserted into every 10 samples to monitor the repeatability of the analysis process.

The coefficient of variation (CV) could reflect the degree of data dispersion. The proportion of substances with CV values less than reference CV values could be analysed by empirical cumulative distribution function (ECDF).

### 4.4. UPLC Conditions [55,56]

The sample extracts were analysed using a UPLC-ESI-MS/MS system (UPLC, SHIMADZU Nexera X2, https://www.shimadzu.com.cn/ (accessed on 18 April 2022); MS, Applied Biosystems 4500 Q TRAP, https://www.thermofisher.cn/cn/zh/home/brands/applied-biosystems.html (accessed on 18 April 2022)).

The analytical conditions were as follows, UPLC: column, Agilent SB-C18 (1.8 µm, 2.1 mm × 100 mm). The mobile phase comprised solvents A (pure water with 0.1% formic acid) and B (acetonitrile with 0.1% formic acid). Sample measurements were performed with a gradient program that employed the starting conditions of 95% A, 5% B. Within 9 min, a linear gradient to 5% A, 95% B was programmed, and a composition of 5% A, 95% B was kept for 1 min. Subsequently, a composition of 95% A, 5.0% B was adjusted within 1.1 min and kept for 2.9 min. The flow velocity was set as 0.35 mL per minute. The column oven was set to 40 °C. The injection volume was 4 μL. The effluent was alternatively connected to an ESI-triple quadrupole-linear ion trap (QTRAP)-MS.

### 4.5. ESI-Q TRAP-MS/MS Analysis [55,56]

Linear ion trap (LIT) and triple quadrupole (QQQ) scans were acquired on a triple quadrupole-linear ion trap mass spectrometer (Q TRAP), AB4500 Q TRAP UPLC/MS/MS System, equipped with an ESI Turbo Ion-Spray interface, operating in positive and negative ion mode and controlled by Analyst 1.6.3 software (AB Sciex, Framingham, MA, USA). The ESI source operation parameters were as follows: ion source, turbo spray; source temperature 550 °C; ion spray voltage (IS) 5500 V (positive ion mode)/-4500 V (negative ion mode); ion source gas I (GSI), gas II(GSII), curtain gas (CUR) were set at 50, 60 and 25.0 psi, respectively; the collision-activated dissociation (CAD) was high. Instrument tuning and mass calibration were performed with 10 and 100 μmol/L polypropylene glycol solutions in QQQ and LIT modes, respectively. QQQ scans were acquired as multiple reaction monitoring (MRM) experiments with collision gas (nitrogen) set to medium. The declustering potential (DP) and collision energy (CE) for individual MRM transitions were done with further DP and CE optimization. A specific set of MRM transitions were monitored for each period according to the metabolites eluted within this period.

### 4.6. Qualitative and Semi-Quantitative Analysis of Metabolites [55,56]

The identification and structural analyses of the primary and secondary spectral data of the metabolites detected by mass spectrometry were based on the MWDB database (Metware Biotechnology Co., Ltd. Wuhan, China) and public databases, including MassBank, KNAPSAcK, HMDB, MoToDB, and ChemBank, PubChem, NIST Chemistry Webbook and METLIN. Metabolomics data were processed using Analyst (Version 1.6.3, Sciex, Framingham, MA, USA).

Metabolite quantification was performed using MRM mode of QQQ mass spectrometry. In the MRM mode, the quadrupole filters the precursor ions of the target substance and excludes the ions corresponding to other molecular weights to eliminate interference. Peak area integration was performed using MultiQuant version 3.0.2 (AB SCIEX, Concord, ON, Canada) after obtaining the metabolite mass spectrometry data. Finally, the chromatographic peak area was used to determine the relative metabolite contents.

### 4.7. Principal Component Analysis [55,56]

Unsupervised principal component analysis (PCA) was performed by statistics function prcomp within R (www.r-project.org (accessed on 18 April 2022)). The data were unit variance scaled before unsupervised PCA.

### 4.8. Hierarchical Cluster Analysis and Pearson Correlation Coefficients [55,56]

The hierarchical cluster analysis (HCA) results of samples and metabolites were presented as heatmaps with dendrograms, while Pearson correlation coefficients (PCC) between samples were calculated by the cor function in R and presented as only heatmaps. Both HCA and PCC were carried out by R package Complex Heatmap. For HCA, normalized signal intensities of metabolites (unit variance scaling) are visualized as a colour spectrum.

### 4.9. Differential Metabolites Selected [55,56]

Significantly regulated metabolites between groups were determined by variable importance in project (VIP) ≥1 and absolute log_2_ FC (fold change) ≥1. VIP values were extracted from Orthogonal Partial Least Squares-Discriminant Analysis (OPLS-DA) results, which also contain score plots and permutation plots, were generated using R package MetaboAnalystR (https://github.com/xia-lab/MetaboAnalystR (accessed on 18 April 2022)). The data were log transformed (log_2_) and mean cantered before OPLS-DA. In order to avoid overfitting, a permutation test (200 permutations) was performed.

### 4.10. KEGG Annotation and Enrichment Analysis [55,56]

Identified metabolites were annotated using KEGG Compound database (http://www.kegg.jp/kegg/compound/ (accessed on 18 April 2022)), and annotated metabolites were then mapped to KEGG Pathway database (http://www.kegg.jp/kegg/pathway.html (accessed on 18 April 2022)). Pathways with significantly regulated metabolites mapped to were then fed into metabolite sets enrichment analysis (MSEA), and their significance was determined by hypergeometric test’s *p*-values.

## 5. Conclusions

This study used the methods of widely targeted metabolomics analysis, and the detection methods were stable and reliable. Taking representative soybean (Heihe 43) and chickpea (Muying No.1) as research objects, we systematically identified and relatively quantified more than 10 kinds of metabolites, with a total of 776 metabolites. It was also found that the stability of metabolites accumulation in soybean was lower than that in chickpea. Among these metabolites, 480 metabolites showed significant differences between chickpea and soybean. Most of these DAMs were concentrated in four types of compounds, including flavonoids (24.20%), lipids (15.20%), amino acids and derivatives (10.40%) and phenolic acids (10.00%). The relative contents of 106 metabolites in chickpea were up-regulated compared with that in soybean. The relative contents of 374 metabolites in soybean were up-regulated compared with that in chickpea. This finding provided important basic data for future research on chickpea and soybean. The differences in hypoglycaemic activity between soybean and chickpea and their applicability were also analysed on the basis of the metabolite data of lipids, saccharides, isoflavones and triterpene saponins. It was found that chickpea was more suitable for modern people to play the role of auxiliary hypoglycaemic health care. In addition, many novel hypoglycaemic compounds had been found in chickpea, some of which had much higher contents than those in soybean, or only exist in chickpea, which provided a basis for further research and development of chickpea. It also provided a reference for the development of new hypoglycaemic lead compounds. Through KEGG pathway, the differential metabolites of soybean and chickpea were found to be concentrated in the metabolic pathways of isoflavonoid biosynthesis, flavonoid biosynthesis, flavone and flavonol biosynthesis, phenylpropanoid biosynthesis, linoleic acid metabolism, and tropane, piperidine and pyridine alkaloid biosynthesis. These metabolic pathways played an important role in regulating the healthcare functions of the two legumes. This study provided an important reference for further research and development of chickpea and soybean based on these abundant data.

## Figures and Tables

**Table 1:** Overview of the identified metabolites in DD and YZD.

Primary Classification (Total)	Secondary Classification	Number of Metabolites
Lipids (130)	Free fatty acids	67
	Lyso-Phosphatidylcholines	32
	Lyso-Phosphatidyl ethanolamines	23
	Glycerol esters	7
	Phosphatidylcholines	1
Flavonoids (124)	Isoflavones	41
	Flavonols	27
	Flavones	25
	Flavanones	15
	Flavonoid carbonside	6
	Chalcones	6
	Flavanonols	2
	Dihydroisoflavones	1
	Flavonols	1
Amino acids and derivatives (90)	Amino acids and derivatives	90
Phenolic acids (85)	Phenolic acids	85
Organic acids (68)	Organic acids	68
Nucleotides and derivatives (57)	Nucleotides and derivatives	57
Alkaloids (56)	Alkaloids	21
	Plumerane	16
	Phenolamine	8
	Piperidine alkaloids	3
	Pyridine alkaloids	2
	Pyrrole alkaloids	2
	Quinorisidine alkaloids	2
	Isoquinoline alkaloids	1
	Quinoline alkaloids	1
Terpenoids (43)	Triterpene Saponin	25
	Triterpene	10
	Sesquiterpenoids	3
	Monoterpenoids	3
	Ditepenoids	2
Lignans and Coumarins (17)	Lignans	10
	Coumarins	7
Quinones (10)	Anthraquinone	8
	Quinones	2
Others (96)	Saccharides and Alcohols	65
	Vitamins	12
	Others	19

**Table 2:** Overview of the differentially accumulated metabolites in potential anti-diabetic components between YZD and DD.

Primary Classification	Secondary Classification	DD vs. YZD	
		Down	Up
Lipids	Free fatty acids	28	6
	Glycerol ester	7	0
	Lyso-Phosphatidylcholines	18	1
	Lyso-Phosphatidyl ethanolamines	13	0
Saccharides	Saccharides	19	4
Flavonoids	Chalcones	6	0
	Dihydroisoflavones	1	0
	Flavanols	1	0
	Flavanones	14	1
	Flavanonols	2	0
	Flavones	17	7
	Flavonoid carbonoside	5	1
	Isoflavones	32	8
	Flavonols	10	11
Terpenoids	Monoterpenoids	3	0
	Sesquiterpenoids	1	0
	Triterpene	8	0
	Triterpene Saponin	21	1

## Data Availability

The data presented in this study are available in article and Supplementary Material.

## Supplementary Materials

The following supporting information can be downloaded at: https://www.mdpi.com/article/10.3390/molecules27165297/s1, Figure S1: QC MS TIC-N (Negative ion mode); Figure S2: QC MS TIC-P (Positive ion mode); Figure S3: DD vs. YZD OPLS-DA model validation; Figure S4: DD vs YZD KEGG barplot; Table S1: List and characteristics of the metabolites identified and quantified in DD and YZD; Table S2: List of DAMs in the YZD vs. DD; Table S3: Statistics of DAMs between YZD and DD; Table S4: List of KEGG pathway in the YZD vs. DD.

## Sample Availability

Samples of the compounds are not available from the authors.
