# Supplementary figures and images for: Widely Targeted Metabolomics Analysis of Soybean and Chickpea and Their Different Advantages and New Functional Compounds for Diabetes
Source: Molecules. 2022 Aug 19;27(16):5297. doi: 10.3390/molecules27165297 (PMC9413387; doi:10.3390/molecules27165297)

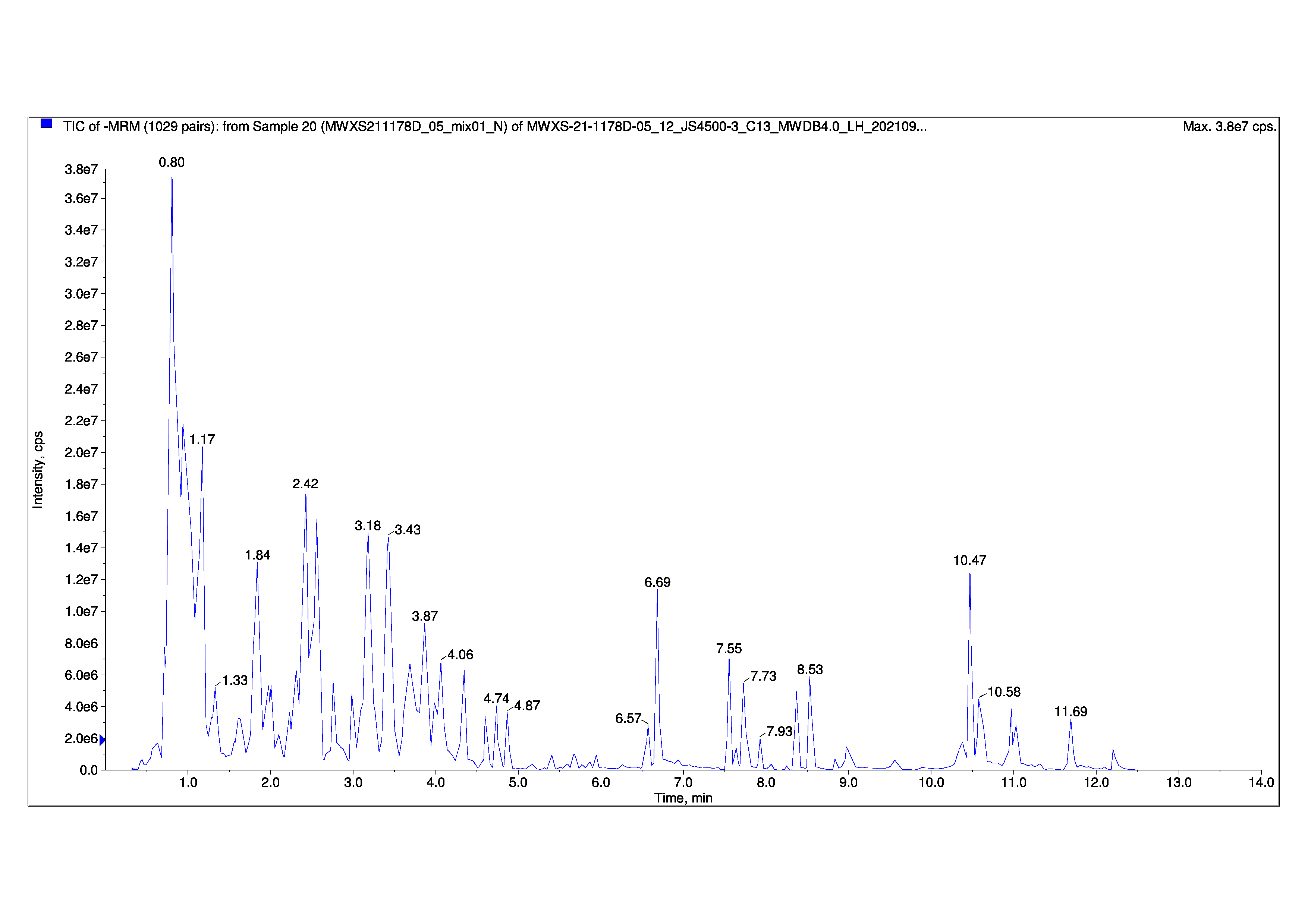

Supplement: Supplementary file 1 [file molecules-27-05297-s001.zip › Supplementary Files/Supplementary Figures/Figure S1_QC_MS_TIC-N(Negative ion mode).png]

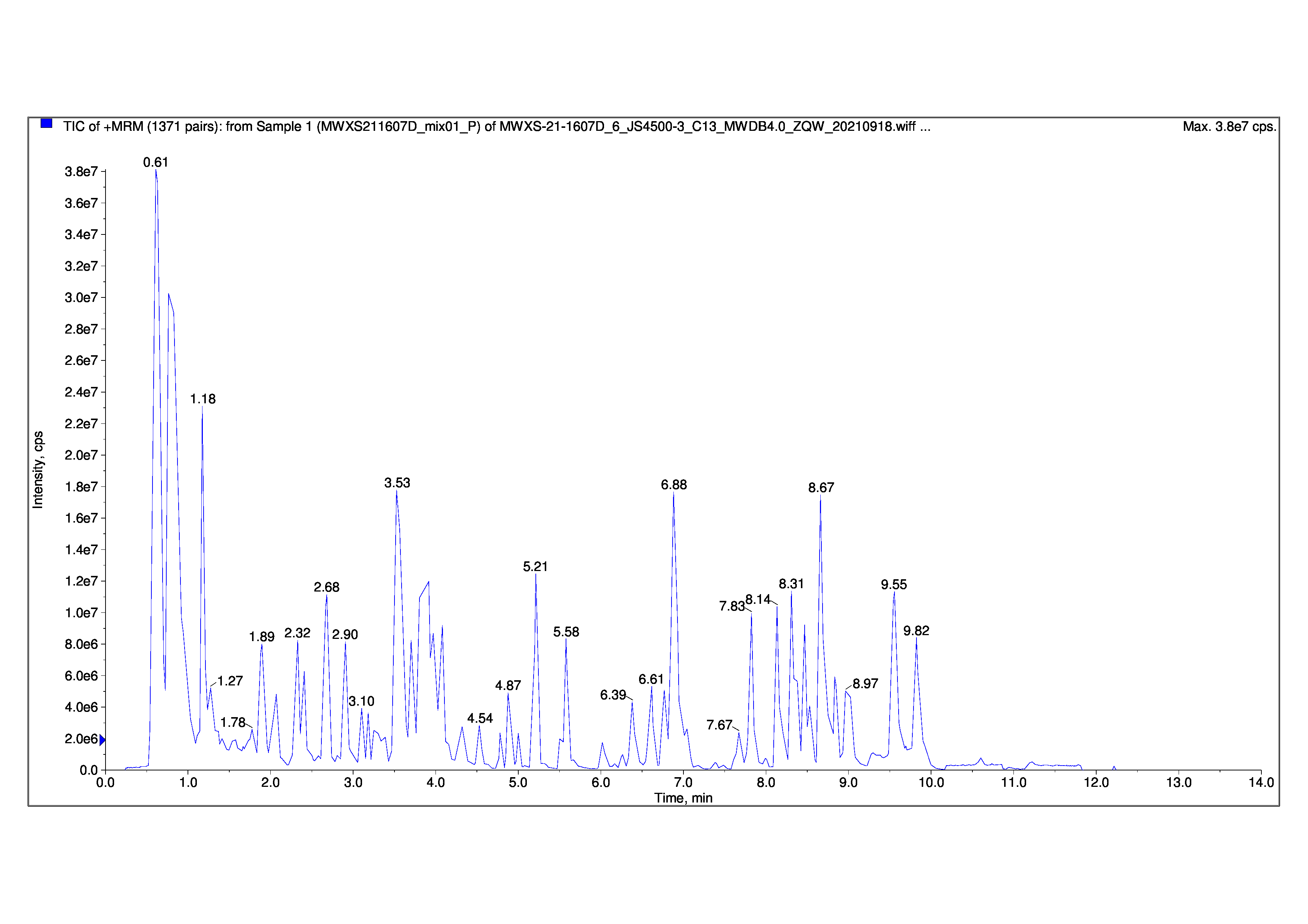

Supplement: Supplementary file 1 [file molecules-27-05297-s001.zip › Supplementary Files/Supplementary Figures/Figure S2_QC_MS_TIC-P(Positive ion mode).png]

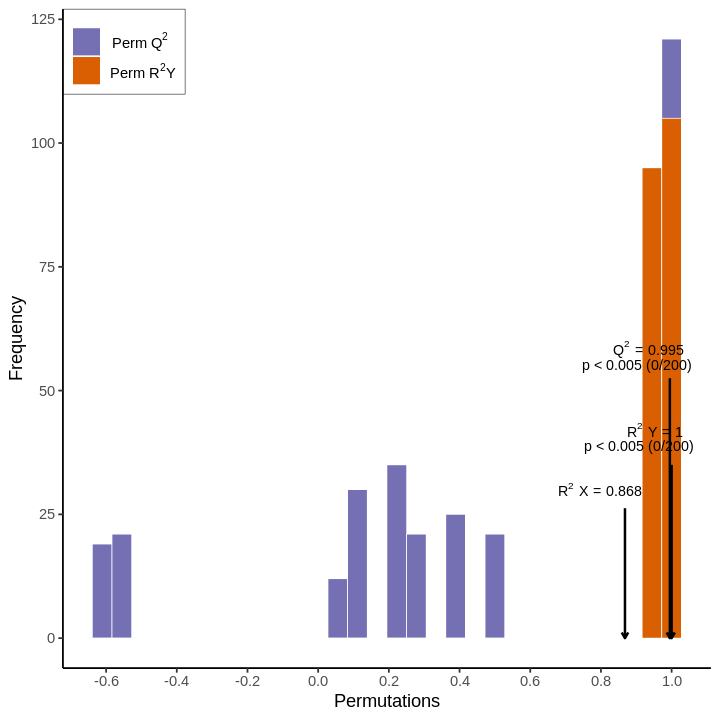

Supplement: Supplementary file 1 [file molecules-27-05297-s001.zip › Supplementary Files/Supplementary Figures/Figure S3_DD_vs_YZD_OPLS_DA_model validation.png]

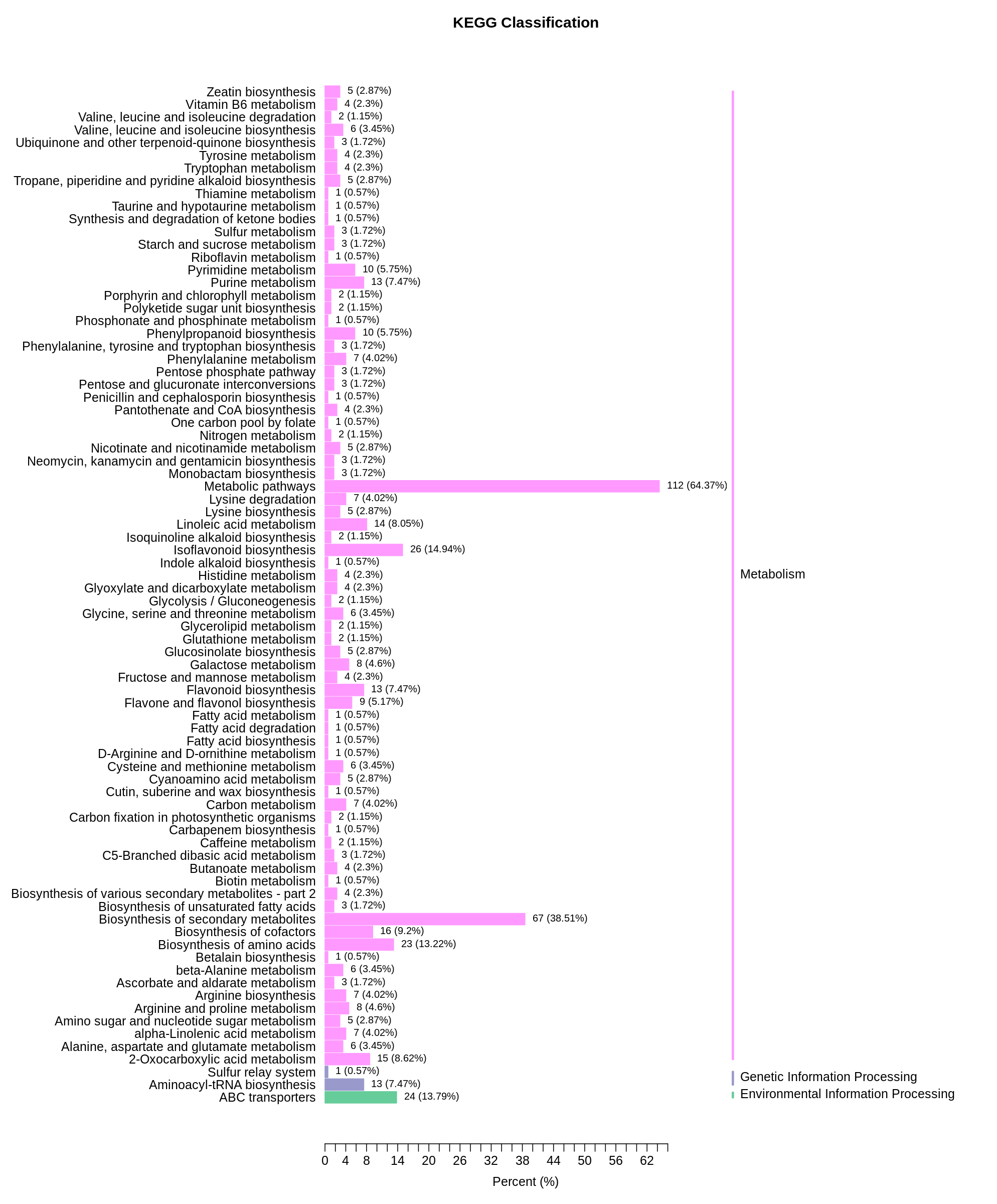

Supplement: Supplementary file 1 [file molecules-27-05297-s001.zip › Supplementary Files/Supplementary Figures/Figure S4_DD_vs_YZD.KEGG.barplot.png]
